# Enhanced abundance and activity of the chloroplast ATP synthase in rice through the overexpression of the AtpD subunit

**DOI:** 10.1093/jxb/erac320

**Published:** 2022-07-29

**Authors:** Maria Ermakova, Eiri Heyno, Russell Woodford, Baxter Massey, Hannah Birke, Susanne von Caemmerer

**Affiliations:** Centre of Excellence for Translational Photosynthesis, Division of Plant Science, Research School of Biology, The Australian National University, Canberra, Australian Capital Territory, Australia; Centre of Excellence for Translational Photosynthesis, Division of Plant Science, Research School of Biology, The Australian National University, Canberra, Australian Capital Territory, Australia; Centre of Excellence for Translational Photosynthesis, Division of Plant Science, Research School of Biology, The Australian National University, Canberra, Australian Capital Territory, Australia; Centre of Excellence for Translational Photosynthesis, Division of Plant Science, Research School of Biology, The Australian National University, Canberra, Australian Capital Territory, Australia; Centre of Excellence for Translational Photosynthesis, Division of Plant Science, Research School of Biology, The Australian National University, Canberra, Australian Capital Territory, Australia; Centre of Excellence for Translational Photosynthesis, Division of Plant Science, Research School of Biology, The Australian National University, Canberra, Australian Capital Territory, Australia; Lancaster University, UK

**Keywords:** ATP synthase, CO_2_ assimilation, electron transport, photosynthesis, proton motive force, thylakoid membrane

## Abstract

ATP, produced by the light reactions of photosynthesis, acts as the universal cellular energy cofactor fuelling all life processes. Chloroplast ATP synthase produces ATP using the proton motive force created by solar energy-driven thylakoid electron transport reactions. Here we investigate how increasing abundance of ATP synthase affects leaf photosynthesis and growth of rice, *Oryza sativa* variety Kitaake. We show that overexpression of AtpD, the nuclear-encoded subunit of the chloroplast ATP synthase, stimulates both abundance of the complex, confirmed by immunodetection of thylakoid complexes separated by Blue Native-PAGE, and ATP synthase activity, detected as higher proton conductivity of the thylakoid membrane. Plants with increased AtpD content had higher CO_2_ assimilation rates when a stepwise increase in CO_2_ partial pressure was imposed on leaves at high irradiance. Fitting of the CO_2_ response curves of assimilation revealed that plants overexpressing AtpD had a higher electron transport rate (*J*) at high CO_2_, despite having wild-type-like abundance of the cytochrome *b*_6_*f* complex. A higher maximum carboxylation rate (*V*_cmax_) and lower cyclic electron flow detected in transgenic plants both pointed to an increased ATP production compared with wild-type plants. Our results present evidence that the activity of ATP synthase modulates the rate of electron transport at high CO_2_ and high irradiance.

## Teaser

Overexpression of the AtpD subunit of the chloroplast ATP synthase in rice increases the abundance and activity of the complex and stimulates photosynthetic electron transport at high light and high CO_2_.

## Introduction

The efficiency of light interception is one of the major factors affecting crop yield (Zhu *et al.*, 2010), and its improvement presents a promising route for increasing plant productivity (Evans, 2013; Long *et al.*, 2015; Walter and Kromdijk, 2022). Absorption of light and conversion of light into chemical energy are performed by the light reactions of photosynthesis which are localized to the thylakoid membranes of chloroplasts. Using the energy of light, electrons originating from water split by PSII are transferred by the chain of cofactors to PSI to reduce NADP^+^. This process is known as linear electron flow (LEF). Between the two photosystems, the cytochrome *b*_6_*f* complex (Cyt*b*_6_*f*) links electron transport with the translocation of protons across the thylakoid membrane, from stroma to lumen, which establishes the electrochemical proton gradient termed the proton motive force (*pmf*). The latter is used by the ATP synthase complex to produce ATP from ADP and P_i_. During the so-called dark reactions of photosynthesis, CO_2_ is fixed into sugars by enzymes of the photosynthetic carbon reduction cycle which requires a minimum of 3 ATP and 2 NADPH to fix 1 CO_2_. Since the chloroplast ATP synthase needs 4.7 protons to produce 1 ATP (von Ballmoos *et al.*, 2008), LEF only results in production of 2.6 ATP molecules per 2 NADPH. To achieve a ratio of 3 ATP to 2 NADPH and to supply ATP for other metabolic processes, plants run cyclic electron flow (CEF) around PSI that establishes additional *pmf* and thus increases ATP production (Joliot and Joliot, 2005).

The capacity of the photosynthetic light reactions must be closely adjusted to their metabolic consumption by the photosynthetic carbon reduction cycle and other anabolic pathways (Cruz *et al.*, 2005; Walker *et al.*, 2020). Therefore, ATP synthase activity is tightly controlled by multilevel regulatory systems providing feedback from both light and dark reactions (Kanazawa *et al.*, 2017). Redox modulation of ATP synthase through the thioredoxin system stimulates its activity under low light and in response to dark–light transition (Carrillo *et al.*, 2016; Kohzuma *et al.*, 2017). At low CO_2_, the activity of ATP synthase is rapidly inhibited (Kanazawa and Kramer, 2002), which allows a build-up of the transmembrane proton gradient, a major component of *pmf*, that triggers non-photochemical quenching (NPQ) (Takizawa *et al.*, 2007). The latter is a suite of photoprotective reactions aimed at reducing the excitation energy reaching the reaction centres of PSII by dissipating a part of absorbed light as heat in the PSII antennae (Malnoë, 2018). PROTON GRADIENT REGULATION5 (PGR5), mediating one of the CEF routes, cooperates with ATP synthase in building up the proton gradient to up-regulate NPQ (Ruban, 2016; Yamori and Shikanai, 2016).

ATP synthase is comprised of nine subunits organized in two major subcomplexes, CF_0_ and CF_1_. The membrane-integral CF_0_ subcomplex consists of four subunits (a, b, bʹ, and c) and the extrinsic CF_1_ subcomplex is made up of five subunits (α, β, γ, δ, and ε) (Capaldi and Aggeler, 2002; Hahn *et al.*, 2018). ATP synthesis in CF_1_ is powered by the CF_0_ rotary motor in the membrane. In *Arabidopsis thaliana* (Arabidopsis), the bʹ, δ, and γ subunits are encoded by the nuclear genes *atpG*, *atpD*, and *atpC1*/*C2*, respectively. The δ subunit (hereafter AtpD) plays an important role in stabilizing the structure of the complex and regulating ATP synthesis because it forms a peripheral stalk together with subunits b and bʹ that acts as a stator to prevent unproductive rotation of CF_1_ with CF_0_ (Maiwald *et al.*, 2003; Hahn *et al.*, 2018). Plants with reduced AtpD abundance show decreased LEF and increased NPQ (Price *et al.*, 1995; Yamori *et al.*, 2011).

Rice (*Oryza sativa*) is one of the world’s staple crops, and substantial efforts are being made towards improving rice productivity. The Kitaake variety is often used as a model crop because it is smaller and has a shorter life cycle compared with *indica* varieties, and transformation techniques have been established. Here we test whether overexpression of the AtpD subunit of ATP synthase affects the complex formation and photosynthesis in rice. We show that overexpressing AtpD results in enhanced abundance and activity of ATP synthase and has the potential to be used for photosynthesis improvement.

## Materials and methods

### Construct assembly and transformation

The coding sequence of *O. sativa atpD* (OsKitaake02g334900.1, Phytozome, https://phytozome.jgi.doe.gov/) was codon optimized for rice and the Golden Gate cloning system (Engler *et al.*, 2014) using the IDT online tool (https://sg.idtdna.com/CodonOpt) and translationally fused at the 3ʹ end with the glycine linker and the Myc-tag-coding sequence (EQKLISEEDL). The resulting sequence was assembled with the *Zea mays Ubiquitin1* promoter and the bacterial *Nos* terminator into the second expression module of the pAGM4723 binary vector. The first module of the binary vector contained the coding sequence of the hygromycin phosphotransferase gene (*hpt*) combined with the *O. sativa Actin1* promoter and *Nos* terminator. The construct was transformed into *O. sativa* ssp. *japonica* variety Kitaake using *Agrobacterium tumefaciens* strain AGL1 following the procedure described in Ermakova *et al.* (2021). T_0_ plants resistant to hygromycin were transferred to soil and analysed for the presence of AtpD-Myc by immunoblotting with Myc antibodies and for the *hpt* copy number by digital PCR (iDNA Genetics, Norwich, UK). Lines 2, 9, and 15 were selected for further analysis based on a stronger AtpD-Myc signal from immunoblots per transgene insertion number and the availability of seeds. Homozygous T_2_ seeds were obtained by selfing T_0_ and T_1_ plants and then used in all experiments unless stated otherwise. Wild-type (WT) plants were used as control in all experiments.

### Plant growth conditions

Plants were grown in a controlled-environment chamber (Model PGC Flex, Conviron, Winnipeg, Canada) under ambient CO_2_ partial pressure (*p*CO_2_), 16 h photoperiod, 28 °C day, 22 °C night, and 60% humidity. Irradiance at 400 μmol photons m^−2^ s^−1^ was supplied by a mixture of Pentron Hg 4 ft fluorescent tubes (54 W 4100 K cool white, Sylvania, Wilmington, MA, USA) and halogen incandescent globes (42 W 2800 K warm white clear glass 630 lumens, CLA, Brookvale, Australia). Plants were grown in 1.2 litre pots in a potting mix composed of 80% peat/10% perlite/10% vermiculite (pH 5.6–5.8) mixed with 5 g l^−1^ of slow-release fertilizer (Osmocote, Evergreen Garden Care, Australia). All pots were kept at field water capacity. All measurements were performed on the mid-distal leaf blade portions of the youngest fully expanded leaves from the central stem of 4-week-old plants.

### Gas exchange

Gas exchange and fluorescence analyses were performed using a LI-6800 (LI-COR Biosciences, Lincoln, NE, USA) equipped with a fluorometer head 6800-01A (LI-COR Biosciences). Leaves were first equilibrated at 381 µbar *p*CO_2_ in the reference side, leaf temperature 25 °C, 60% humidity, flow rate of 500 µmol s^−1^, and 1500 µmol photons m^−2^ s^−1^ (90% red/10% blue actinic light). For the CO_2_ response curves, a stepwise increase of *p*CO_2_ from 0 to 1525 µbar was imposed at 3 min intervals. The maximum carboxylation rate of Rubisco (*V*_cmax_), the rate of electron transport (*J*), and triose phosphate usage (TPU) were obtained using the fitting routine of Sharkey *et al.* (2007). Leaf mesophyll conductance to CO_2_ of 0.67 µmol CO_2_ m^−2^ s^−1^ bar^−1^ previously determined for rice (von Caemmerer and Evans, 2015) was used in the fitting routine. The CO_2_ compensation point was calculated from the CO_2_ response curves recorded at different O_2_ partial pressures (*p*O_2_). Light–response curves were measured during a stepwise decrease of irradiance from 2000 µmol m^−2^ s^−1^ to 0 µmol m^−2^ s^−1^ at 3 min intervals and at 381 µbar *p*CO_2_ in the reference side. The quantum yield of PSII was calculated at different irradiances and *p*CO_2_ upon the application of a multiphase saturating pulse (8000 µmol m^−2^ s^−1^) according to Genty *et al.* (1989).

### Protein isolation and western blotting

Total protein extracts were isolated from 0.5 cm^2^ frozen leaf discs and separated by SDS–PAGE according to Ermakova *et al.* (2019). Proteins were transferred to a nitrocellulose membrane and probed with antibodies against various photosynthetic proteins in dilutions recommended by the manufacturer: Rieske (AS08 330, Agrisera, Vännäs, Sweden), AtpH (Agrisera, AS09591), Myc-tag (ab9132, Abcam, Cambridge, UK), whole ATP synthase (Agrisera, AS08370), D1 (Agrisera, AS10 704), and PGR5 (Agrisera, AS163985). Quantification of immunoblots was performed with Image Lab software (Biorad, Hercules, CA, USA).

### Thylakoid isolation and Blue Native-PAGE

Thylakoid membranes from the mid portions of the three youngest fully expanded leaves collected from one plant were ground in 100 ml of ice-cold grinding buffer (50 mM HEPES-NaOH, pH 7.5, 330 mM sorbitol, 5 mM MgCl_2_) in an Omni Mixer (Thermo Fisher Scientific, Tewksbury, MA, USA). The homogenate was passed through a double layer of Miracloth (Merck Millipore, Burlington, MA, USA) and centrifuged at 6000 rpm, 4 °C for 5 min. Pellets were first resuspended in ice-cold shock buffer (50 mM HEPES-NaOH, pH 7.5, 5 mM MgCl_2_) and centrifuged again. The resulting pellets were resuspended in ice-cold storage buffer (50 mM HEPES-NaOH, pH 7.5, 100 mM sorbitol, 10 mM MgCl_2_) and centrifuged again. Finally, pellets were resuspended in an equal aliquot of the storage buffer, snap-frozen in liquid N_2_, and stored at −80 °C. Preparation of the samples and Blue Native-PAGE followed Rantala *et al.* (2018). The gel was scanned, then incubated for 30 min in the transfer buffer (25 mM Tris, 25 mM glycine, 20% methanol, 0.1% SDS) and blotted to a nitrocellulose membrane. Western blotting was then performed as usual.

### Electron transport and electrochromic shift analyses

To obtain PSII parameters (PhiPSII, the effective quantum yield; PhiNPQ, the yield of non-photochemical quenching; PhiNO, the yield of non-regulated non-photochemical quenching) and PSI parameters (PhiPSI, the effective quantum yield; PhiND, the non-photochemical yield caused by donor side limitation; PhiNA, the non-photochemical yield caused by the acceptor side limitation), fluorescence analysis was performed simultaneously with the spectroscopic measurements at 820 nm using the Dual-PAM-100 (Heinz Walz, Effeltrich, Germany). Measurements were done using red actinic light and 300 ms saturating pulses of 10 000 µmol m^−2^ s^−1^. Leaves were dark-adapted for 30 min to record the minimal and maximal levels of fluorescence in the dark. Then a saturating pulse was given after pre-illumination with far-red light to record the maximal and minimal oxidation levels of P700 (the reaction centre of PSI). To allow for a brief photoactivation, the leaves were next illuminated for 8 min with actinic light of 378 µmol m^−2^ s^−1^ and briefly dark-adapted again for 2 min. After that, photosynthetic parameters were assessed over a range of irradiances from 0 to 2043 µmol m^−2^ s^−1^ at 2 min intervals by applying a saturating pulse at the end of each illumination period. The parameters were calculated according to Kramer *et al.* (2004) and Klughammer and Schreiber (2008). The kinetics of P700 oxidation upon the change of light intensity presented in Fig. 5 were extracted from these measurements.

The electrochromic shift (ECS) signal was monitored as the absorbance change at 515–550 nm using the Dual-PAM-100 equipped with the P515/535 emitter–detector module (Heinz Walz). Leaves were first dark adapted for 40 min, and the absorbance change induced by a single turnover flash was measured. Dark interval relaxation of the ECS signal was recorded after 3 min of illumination with red actinic light of increasing irradiance. Proton conductivity of the thylakoid membrane through the ATP synthase was calculated as an inverse of the time constant obtained by fitting the first-order ECS relaxation (Sacksteder and Kramer, 2000). Total *pmf* was estimated from the amplitude of the rapid decay of the ECS signal normalized for ECS signal change induced by the single turnover flash.

### Chlorophyll, protein, and Rubisco active sites

Frozen leaf discs were ground using the Qiagen TissueLyser II (Qiagen, Venlo, The Netherlands) and total Chl (*a*+*b*) was extracted in 80% acetone buffered with 25 mM HEPES-KOH and measured according to Porra *et al.* (1989). The amount of Rubisco sites was assayed by [^14^C]carboxyarabinitol bisphosphate binding as described in Ruuska *et al.* (2000). Total protein content was measured from the same samples by Coomassie Plus protein assay reagent (Thermo Fisher Scientific).

### Statistical analysis

For all measurements, the relationship between mean values of transgenic and WT plants was tested using heteroscedastic Student’s *t*-tests.

## Results

### Overexpression of AtpD increases abundance of ATP synthase

**Fig. 1.**
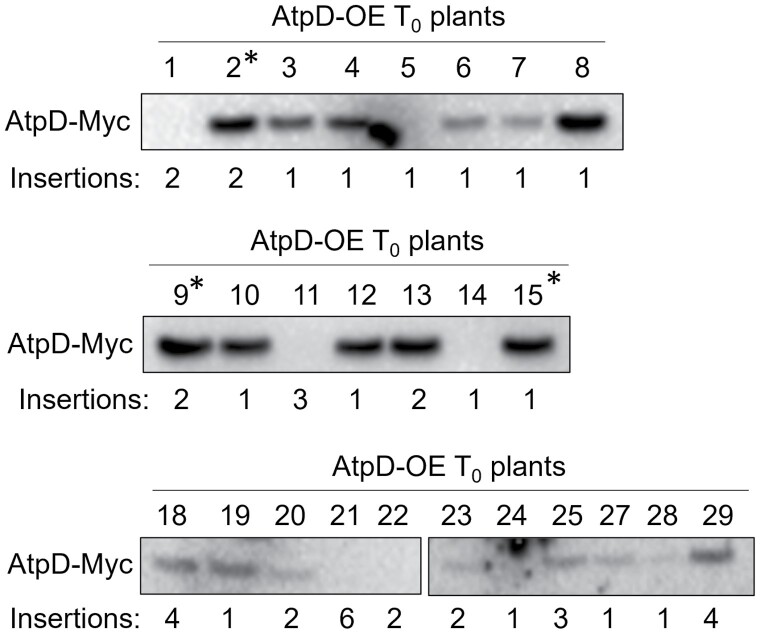
Selection of T_0_ rice plants transformed with the construct for AtpD overexpression (AtpD-OE). Immunodetection of AtpD-Myc in leaf protein extracts, 15 µg of protein loaded for each sample. The copy number of the hygromycin phosphotransferase gene (*hpt*) detected by digital PCR was used to estimate the number of construct insertions. *Lines 2, 9, and 15 were selected for further experiments.

The gene construct for AtpD overexpression (AtpD-OE hereafter) was transformed into rice calli, and T_0_ plants resistant to hygromycin were selected and transferred to soil. T_0_ plants were analysed for the presence of AtpD-Myc protein and for gene insertions based on the *hpt* copy number (Fig. 1). Out of 26 T_0_ plants, 19 plants showed detectable levels of AtpD-Myc. Lines 2, 9, and 15 were selected for further analysis, and homozygous T_2_ plants were studied in all experiments.

**Fig. 2.**
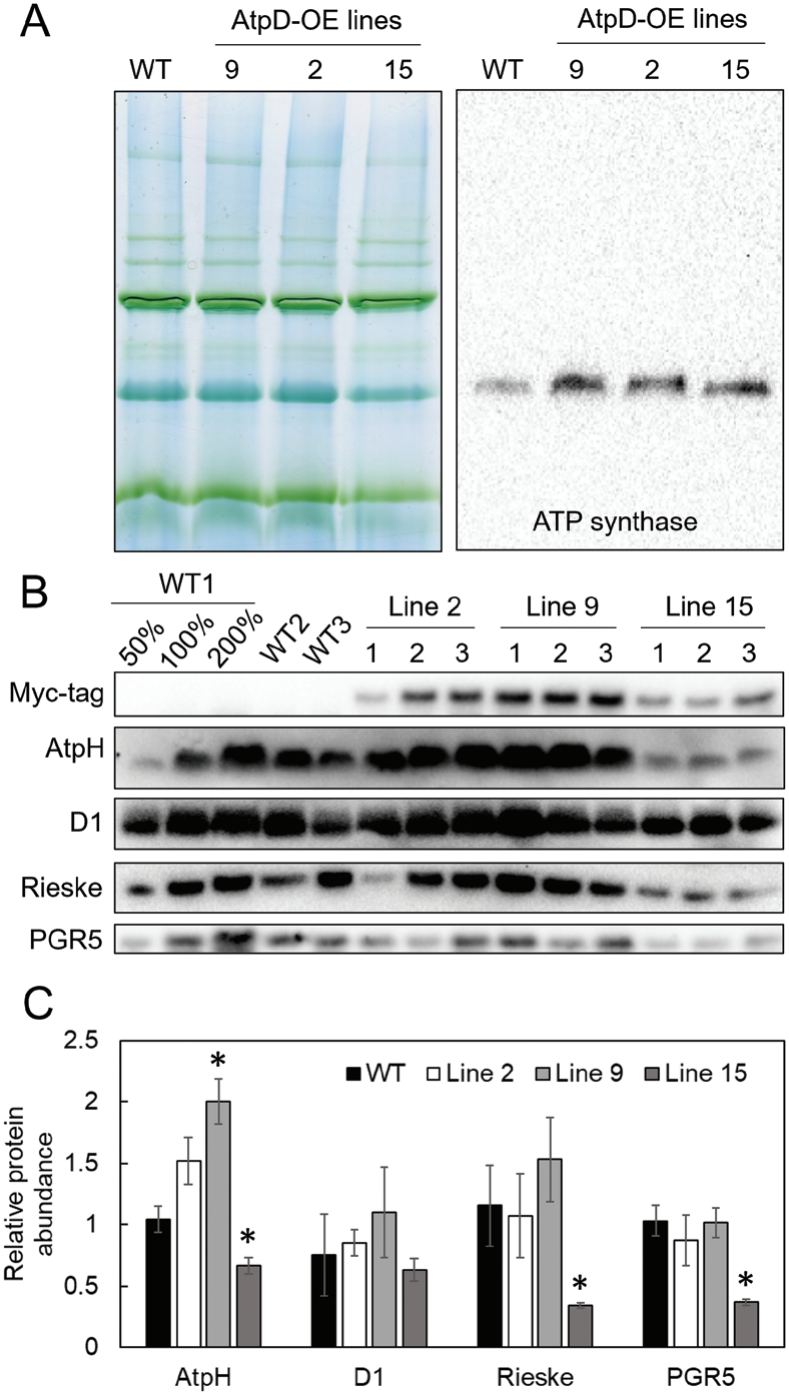
Immunodetection of photosynthetic proteins in WT *O. sativa* and transgenic lines overexpressing AtpD. (A) Thylakoid protein complexes separated by Blue Native-PAGE and probed with antibodies against whole ATP synthase; 10 µg of Chl (*a*+*b*) loaded in each lane. (B) Leaf protein samples loaded on a leaf area basis and probed with antibodies against Myc-tag, AtpH (subunit *c* of ATP synthase), D1 subunit of PSII, Rieske subunit of Cyt *b*_6_*f*, and PGR5 (PROTON GRADIENT REGULATION5). (C) Relative quantification of immunoblots. Mean ±SE, *n*=3 biological replicates. Asterisks indicate statistically significant differences between transgenic lines and the WT (*t*-test, *P*<0.05).

The three selected AtpD-OE lines demonstrated increased abundance of the whole ATP synthase complex in their thylakoid membranes, compared with WT plants, when the samples were normalized on a Chl (*a*+*b*) basis (Fig. 2A). When total leaf protein extracts were loaded on a leaf area basis, lines 2 and 9 had more AtpD-Myc than line 15 and also increased abundance of AtpH, the *c* subunit of the F_0_ complex, compared with WT plants (Fig. 2B). Abundances of D1 (PSII core subunit), Rieske (Cyt*b*_6_*f* core subunit), and PGR5 were unaltered in transgenic lines 2 and 9, whilst line 15 had significantly less AtpH, Rieske, and PGR5, compared with the WT (Fig. 2C). Line 9 had increased leaf Chl content compared with the WT (Table 1). Despite having an increased abundance of ATP synthase on a chlorophyll basis, line 15 contained significantly less chlorophyll per leaf area (Table 1) and was excluded from further physiological analysis.

**Table 1.** Gas exchange and fluorescence parameters of wild-type (WT) and AtpD-OE rice plants

Parameter	WT	Line 2	Line 9	Line 15
Chl (*a*+*b*), mmol m^−2^	0.66 ± 0.06	0.72 ± 0.03	0.86 ± 0.02*	0.40 ± 0.03*
Chl *a*/*b*	4.44 ± 0.04	4.40 ± 0.06	4.50 ± 0.06	4.59 ± 0.05*
Soluble protein, g m^−2^	8.49 ± 0.78	10.23 ± 0.37	12.45 ± 0.35*	5.84 ± 0.58*
*F*_V_/*F*_M_	0.806 ± 0.003	0.799 ± 0.005	0.804 ± 0.003	0.791 ± 0.009
*V*_cmax_, µmol CO_2_ m^−2^ s^−1^	106.8 ± 8.3	133.8 ± 14.5	157.4 ± 12.6*	N/A
*J* (LEF), µmol e^−^ m^−2^ s^−1^	125.1 ± 8.0	153.0 ± 1.7*	166.8 ± 5.1*	N/A
*J*/*V*_cmax_	1.17 ± 0.03	1.17 ± 0.13	1.09 ± 0.11	N/A
TPU, µmol CO_2_ m^−2^ s^−1^	9.02 ± 0.39	10.65 ± 0.08*	11.24 ± 0.40*	N/A
*R*_d_, µmol CO_2_ m^−2^ s^−1^	1.52 ± 0.12	1.32 ± 0.07	1.40 ± 0.13	N/A
*R*_d_/ *V*_cmax_	0.0142 ± 0.0005	0.0100 ± 0.0010*	0.0090 ± 0.0006*	N/A
Rubisco sites, µmol m^−2^	34.1 ± 1.7	35.0 ± 1.9	36.9 ± 1.4	N/A
Rubisco sites/soluble protein	4.02	3.42	2.97	N/A
LMA, g(DW) m^−2^	54.2 ± 2.6	50.5 ± 3.6	53.2 ± 1.4	51 ± 2.2

*F*_V_/*F*_M_, the maximum quantum efficiency of PSII; *V*_cmax_, maximum carboxylation rate allowed by Rubisco; *J*, the rate of photosynthetic electron transport based on NADPH requirement; TPU, triose phosphate use; *R*_d_, dark respiration rate. Mean ±SE, *n*=4 biological replicates. Asterisks indicate statistically significant differences between the WT and transgenic plants (*t*-test, *P*<0.05). N/A, not assessed.

### Increased ATP synthase activity in AtpD-OE plants

**Fig. 3.**
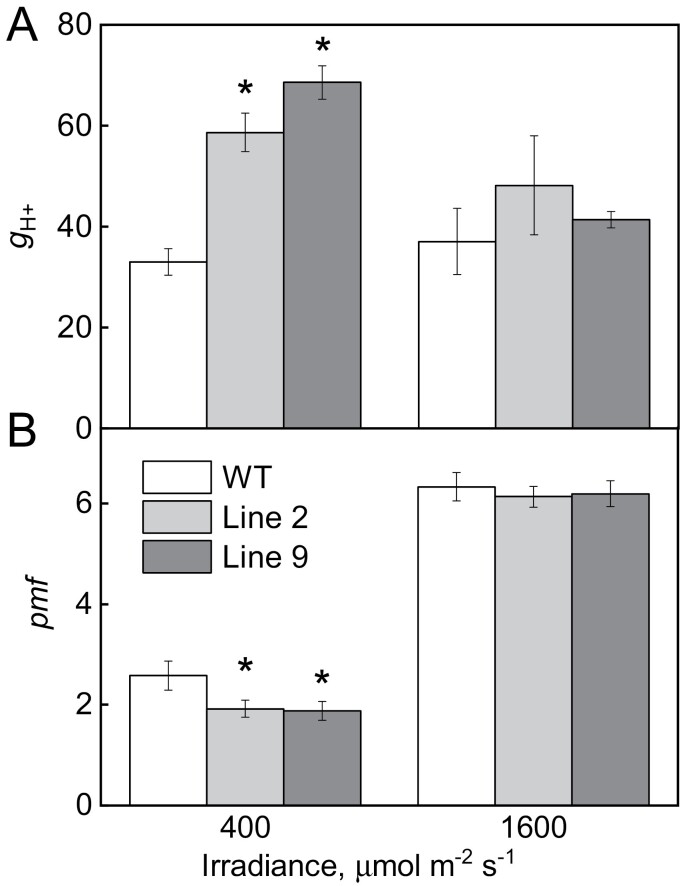
Proton conductivity of the thylakoid membrane (g_H+_) and proton motive force (*pmf*) estimated from the dark interval relaxation kinetics of ECS in WT *O. sativa* and transgenic lines overexpressing AtpD. Mean ±SE, *n*=3-4 biological replicates. Asterisks indicate statistically significant differences between transgenic lines and the WT (*t*-test, *P*<0.05).

Proton conductivity of the thylakoid membrane and *pmf* in leaves of WT and AtpD-OE plants were estimated from the speed and amplitude of ECS relaxation upon the light–dark transition. Lines 2 and 9 showed significantly increased thylakoid proton conductivity at 400 µmol m^−2^ s^−1^, indicating a higher activity of ATP synthase compared with the WT (Fig. 3). When measured at 1600 µmol m^−2^ s^−1^, no significant differences in proton conductivity were detected between the genotypes. In line with that, the amplitude of the fast ECS decay, representing a balance between the build-up and dissipation of *pmf*, was significantly decreased in both AtpD-OE lines at 400 µmol m^−2^ s^−1^ but not at 1600 µmol m^−2^ s^−1^, compared with WT plants (Fig. 3).

### Electron transport properties of AtpD-OE plants

**Fig. 4.**
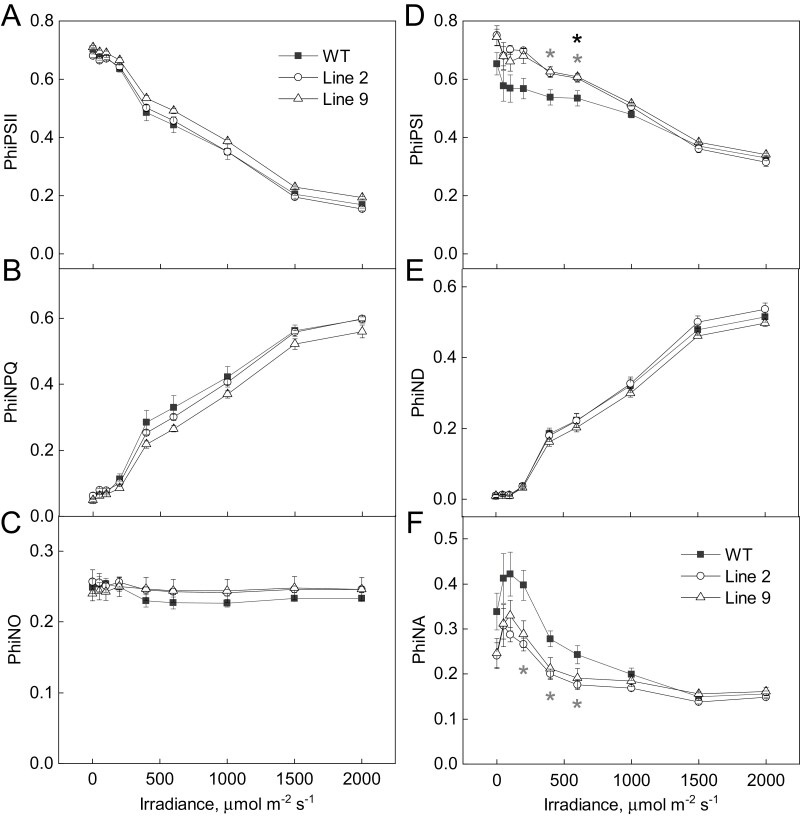
Electron transport properties of WT *O. sativa* and transgenic lines overexpressing AtpD at different irradiances. PhiPSII, quantum yield of PSII; PhiNPQ, quantum yield of non-photochemical quenching; PhiNO, quantum yield of non-regulated non-photochemical quenching; PhiPSI, quantum yield of PSI; PhiND, non-photochemical loss due to the oxidized PSI donors; PhiNA, non-photochemical loss due to the reduced PSI acceptors. Mean ±SE, *n*=3 biological replicates. Grey asterisks indicate statistically significant differences between line 2 and the WT, black asterisks—between line 9 and the WT (*t*-test, *P*<0.05).

**Fig. 5.**
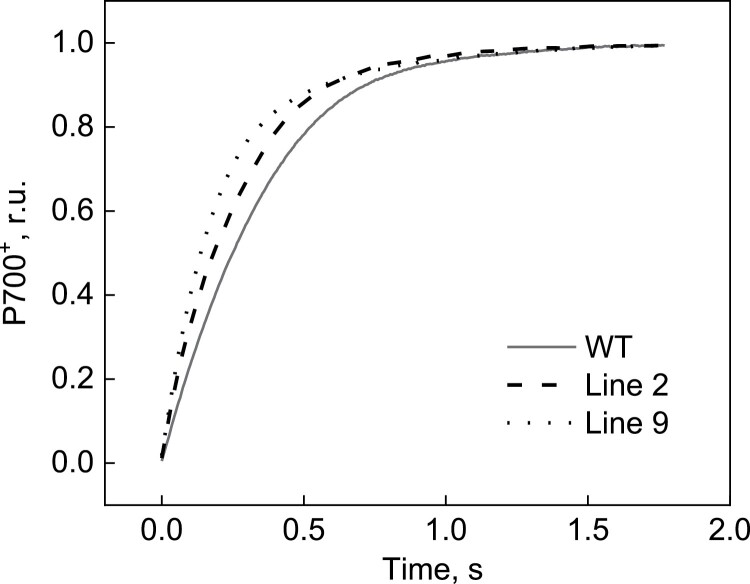
Oxidation kinetics of the reaction centres of PSI (P700) in WT *O. sativa* and transgenic lines overexpressing AtpD during an increase of irradiance from 218 µmol m^−2^ s^−1^ to 417 µmol m^−2^ s^−1^. Curves were normalized to the same amplitude to facilitate comparison of the kinetics and present an average of three biological replicates.

Fluorescence analysis of WT and AtpD-OE plants demonstrated that the maximum quantum efficiency of PSII (*F*_V_/*F*_M_) did not differ between genotypes (Table 1), and neither did the PSII electron transport parameters PhiPSII, PhiNPQ, and PhiNO when measured at different irradiances and ambient *p*CO_2_ (Fig. 4, left panels). Spectroscopic analysis of the redox state of P700, the reaction centre of PSI, at different irradiances (Fig. 4, right panels) showed that the quantum yield of PSI (PhiPSI) was increased in AtpD-OE lines (significant for line 9 at 548 µmol m^−2^ s^−1^ and for line 2 at 417 and 548 µmol m^−2^ s^−1^). The detected increase in PhiPSI in AtpD-OE plants could be attributed to the lower PSI acceptor side limitation (PhiNA), which was significant in line 2 between 218 µmol m^−2^ s^−1^ and 548 µmol m^−2^ s^−1^. The donor side limitation of PSI (PhiND) did not differ between the WT and AtpD-OE plants at any irradiance (Fig. 4, right panels). Further comparison of the kinetics of P700 oxidation during the increase of irradiance from 218 µmol m^−2^ s^−1^ to 417 µmol m^−2^ s^−1^ revealed faster oxidation in AtpD-OE lines, compared with the WT (Fig. 5). This result suggested that PhiNA in AtpD-OE plants was probably reduced due to an increased electron sink capacity downstream of PSI and not due to up-regulated CEF (Joliot and Johnson, 2011).

### Gas exchange analysis of plants with increased ATP synthase abundance

**Fig. 6.**
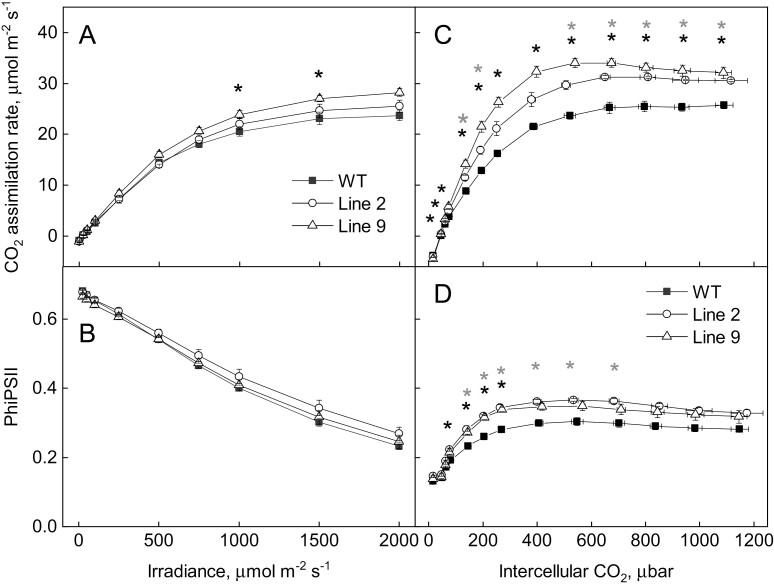
Gas exchange and fluorescence analysis of WT *O. sativa* and transgenic lines overexpressing AtpD. Mean ±SE, *n*=4 biological replicates. Grey asterisks indicate statistically significant differences between line 2 and the WT, black asterisks—between line 9 and the WT (*t*-test, *P*<0.05).

CO_2_ assimilation rates and PhiPSII were assayed in WT and AtpD-OE plants at different irradiances and *p*CO_2_ (Fig. 6). When the light response of photosynthesis was analysed at 381 µbar *p*CO_2_ and high light, line 9 showed significantly increased assimilation rates between 1000 µmol m^−2^ s^−1^ and 1500 µmol m^−2^ s^−1^, compared with the WT (Fig. 6, left panels). The response of CO_2_ assimilation to intercellular *p*CO_2_ (*AC*_i_ curves) was measured at a constant irradiance of 1500 µmol m^−2^ s^−1^ (Fig. 6, right panels). Line 9 had significantly increased assimilation rates at all intercellular *p*CO_2_ except the lowest one, compared with the WT. Line 2 also had significantly increased assimilation rates at ambient and high intercellular *p*CO_2_. Both AtpD-OE lines showed increased PhiPSII, compared with WT plants (significant for line 2 at 133–650 µbar and for line 9 at 74–257 µbar).

**Fig. 7.**
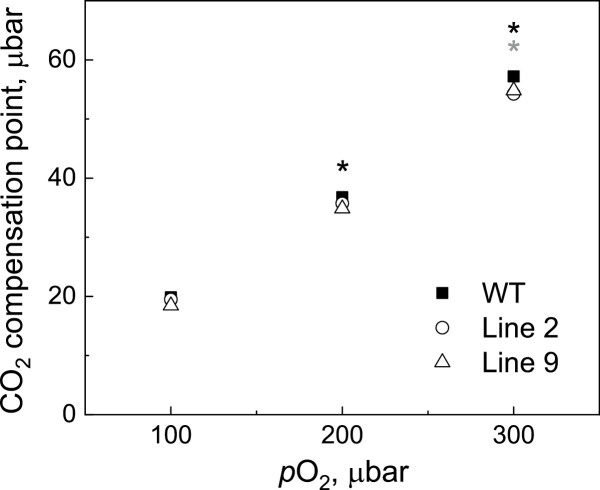
Leaf CO_2_ compensation point measured at different O_2_ partial pressures (*p*O_2_). Mean ±SE, *n*=3 biological replicates; SE is smaller than the symbols. Grey asterisks indicate statistically significant differences between line 2 and the WT, black asterisks—between line 9 and the WT (*t*-test, *P*<0.05).

**Fig. 8.**
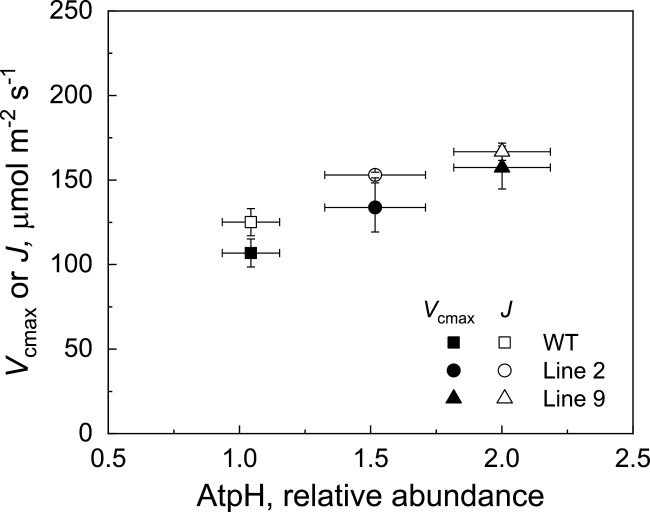
Gas exchange parameters obtained by fitting the CO_2_ response curves of assimilation versus relative protein abundance of AtpH from the immunoblots in Fig. 2.

Fitting of the *AC*_i_ curves revealed significantly increased *J*, the rate of photosynthetic electron transport, and TPU, the triose phosphate use, in AtpD-OE lines 2 and 9, compared with the WT (Table 1). Line 9 also had significantly increased *V*_cmax_, the maximum carboxylation rate allowed by Rubisco (*P*=0.18 for line 2). The *J*/*V*_cmax_ ratio and the rate of respiration in the dark (*R*_d_) did not differ between WT and AtpD-OE plants, whilst the *R*_d_/*V*_cmax_ ratio was significantly lower than in the WT in lines 2 and 9. In line with the observed decrease in *R*_d_/*V*_cmax_, AtpD-OE plants also showed a lower CO_2_ compensation point, significant for line 9 at ambient and high *p*O_2_ and for line 2 at high *p*O_2_ (Fig. 7). Overall, a positive correlation was observed between both *V*_cmax_ and *J* values and ATP synthase abundance (estimated as the relative abundance of the AtpH subunit from immunoblots on Fig. 2) for all three genotypes (Fig. 8). The total abundance of Rubisco active centres measured in the leaves subjected to gas exchange analysis did not differ between WT and AtpD-OE plants, but the total soluble protein content was significantly increased in line 9, compared with the WT (Table 1).

### Biomass and grain yield of AtpD-OE plants

**Fig. 9.**
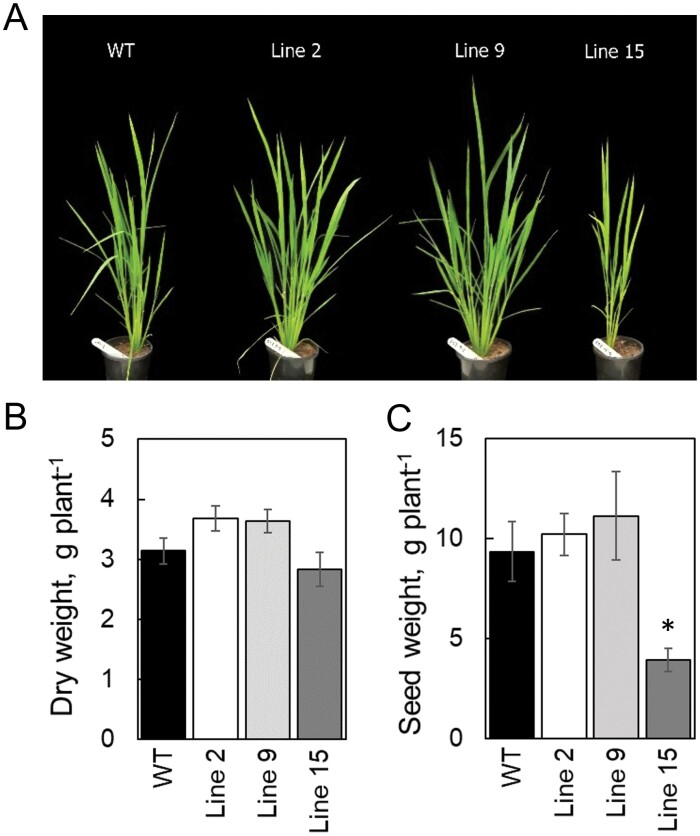
Biomass and seed yield of WT *O. sativa* and transgenic lines overexpressing AtpD. (A) Phenotype of plants during the mid-tillering stage, 4 weeks after germination. (B) Dry weight of the plants harvested at mid-tillering stage, mean ±SE, *n*=8 biological replicates. (C) Total weight of seeds produced by the plants, mean ±SE, *n*=4 biological replicates. Asterisks indicate statistically significant differences between transgenic lines and the WT (*t*-test, *P*<0.05).

Leaf mass per area in AtpD-OE plants was similar to that of the WT (Table 1). During the mid-tillering stage, AtpD-OE plants of lines 2 and 9 were slightly larger than WT plants: *P*=0.097 for line 2 and *P*=0.111 for line 9 (Fig. 9). The total weight of seeds produced by AtpD-OE lines 2 and 9 was similar to that of the WT, whilst plants of line 15 produced significantly fewer seeds (Fig. 9C).

## Discussion

Increasing the rate of electron transfer during the light reactions of photosynthesis is predicted to be of benefit for enhancing crop yield (Simkin *et al.*, 2019). Promising strategies for accelerating electron transport include up-regulating the abundance of Cyt*b*_6_*f* by overexpressing the Rieske subunit to alleviate rate limitation at the step of plastoquinol oxidation (Simkin *et al.*, 2017; Ermakova *et al.*, 2019), speeding up the delivery of electrons to PSI by engineering plants with algal cytochrome *c*_6_ (Chida *et al.*, 2007; López-Calcagno *et al.*, 2020), and facilitating faster relaxation of NPQ (Kromdijk *et al.*, 2016). Although the low productivity of the chloroplast ATP synthase with the high H^+^/ATP ratio of 4.67 was suggested to be favourable for avoiding photodamage (Davis and Kramer, 2020), we were interested to test the effects of increased ATP synthase activity on electron transport. Although the activity of ATP synthase correlates with transcript and protein levels of its subunits (Kohzuma *et al.*, 2009), multiple additional levels of regulation make this complex a regulatory hub collecting signals from light-harvesting reactions, the photosynthetic carbon reduction cycle, and central metabolism (Schöttler and Tóth, 2014).

Rice plants overexpressing the AtpD subunit of ATP synthase demonstrated increased abundance of the whole complex, and two AtpD-OE lines had increased ATP synthase activity detected as higher proton conductivity of the thylakoid membrane (Figs 2, 3). Moreover, two AtpD-OE lines showed a proportional increase in whole-chain electron transport and CO_2_ assimilation rates at high *p*CO_2_ (Table 1; Figs 6, 8). This is in line with the C_3_ photosynthesis model which predicts electron transport limitations at high irradiance and high *p*CO_2_ as well as at lower irradiance (Farquhar and von Caemmerer, 1981; von Caemmerer and Farquhar, 1981). Studies on tobacco plants with reduced *atpD* transcript levels have previously shown a close correlation between CO_2_ assimilation rate and AtpD abundance (Price *et al.*, 1995; Yamori *et al.*, 2011). Given that there are equally close correlations between Rieske content and chloroplast electron transport and CO_2_ assimilation rates, previous works suggested that Cyt*b*_6_*f* and ATP synthase co-limit electron transport (Price *et al.*, 1995; Yamori *et al.*, 2011, 2016). Our results indicate that, at high irradiance and high CO_2_, electron transport is primarily limited by ATP synthase, and the limitation at Cyt*b*_6_*f* could be overcome by increasing AtpD content. At ambient *p*CO_2_, a significant increase of thylakoid proton conductivity was only detected in AtpD-OE plants at growth irradiance but not at high irradiance (Fig. 3), supporting a down-regulation of ATP synthase in conditions when the light reactions are limited by Rubisco activity and metabolic regeneration of NADP^+^, ADP, and P_i_ (Kohzuma *et al.*, 2017).

Curiously, we also observed an increase in *in vivo* Rubisco activity (*V*_cmax_) in two AtpD-OE lines, despite similar amounts of Rubisco in the leaves (Table 1), which matched similar abundances of Rieske and other electron transport components (Fig. 2; Table 1). The lower CO_2_ compensation point detected in two AtpD-OE lines was also in line with the increased *V*_cmax_ and lower *R*_d_/*V*_cmax_ (Table 1; Fig. 7) (Azcon-Bieto and Osmond, 1983; von Caemmerer, 2000). The higher *V*_cmax_ was probably due to the higher activation state of Rubisco in plants overexpressing AtpD. The active sites of Rubisco become inactivated by binding sugar phosphates and require Rubisco activase to restore their activity (Salvucci *et al.*, 1985). The activase is strongly dependent on ATP as it is both regulated by the ATP/ADP ratio and uses ATP for the reaction (Streusand and Portis, 1987; Robinson and Portis, 1988). Higher thylakoid proton conductivity in AtpD-OE plants could provide more ATP for Rubisco activase and sustain Rubisco carboxylation activity in conditions promoting the deactivation, for instance upon exposure to low CO_2_ (von Caemmerer and Edmondson, 1986). Since CEF is strongly inhibited by ATP (Fisher *et al.*, 2019), a lower CEF, seen as a lower PhiNA and faster P700 oxidation kinetics (Figs 4, 5), indicated an increased ATP production in AtpD-OE plants. Because activation of Rubisco is one of the promising traits for improving crop photosynthesis (Parry *et al.*, 2013; Carmo-Silva *et al.*, 2015; Taylor *et al.*, 2022), exploring the relationship between the thylakoid proton conductivity and Rubisco activation could be of great interest for future research.

Importantly, we have developed a method for increasing the abundance and activity of ATP synthase by overexpressing one subunit of the complex. It was previously shown that AtpD is critical for stabilizing the complex and that its abundance correlates with the electron transport rate (Engelbrecht *et al.*, 1989; Price *et al.*, 1995). AtpD is one of the two nuclear gene-encoded subunits of chloroplast ATP synthase. Interestingly, despite the transfer of organellar genes to the nucleus being favourable for the cell energy budget, only a limited number of genes encoding subunits of the thylakoid complexes were translocated to the nucleus; proteins with the highest abundance are more likely to be retained in an organellar genome (Kelly, 2021). This suggests that nuclear-encoded subunits limit the assembly of thylakoid complexes and are involved in retrograde–anterograde signalling pathways integrating chloroplast light reactions with cellular metabolism (Lyska *et al.*, 2013; Pribil *et al.*, 2014). Taking advantage of these signalling pathways provides an opportunity for increasing the abundance of complexes by overexpressing single subunits, as previously reported for Cyt*b*_6_*f* (Simkin *et al.*, 2017; Ermakova *et al.*, 2019) and as shown here for ATP synthase. However, whilst overexpression of Rieske in Arabidopsis allowed higher electron transport and CO_2_ assimilation rates, this was accompanied by the increased abundance of not only Cyt*b*_6_*f* but also ATP synthase subunits, as well as PSII and PSI (Simkin *et al.*, 2017). In contrast, two rice AtpD-OE lines did not display increases in thylakoid membrane complexes other than the ATP synthase and showed a lower *pmf*, suggesting that ATP synthase activity exceeded Cyt*b*_6_*f* activity. Therefore, increasing Cyt*b*_6_*f* abundance could be complementary to AtpD overexpression to further boost the electron transport rate and stimulate photosynthesis.

### Conclusion

Photosynthetic light reactions are tightly regulated to prevent photodamage, but in some conditions overcoming or reducing the regulation of photosynthesis could be of benefit for plant productivity (Kromdijk *et al.*, 2016). Here we show that overexpressing the AtpD subunit of the chloroplast ATP synthase was sufficient to increase abundance of the whole complex. Moreover, two AtpD-OE lines showed increased ATP synthase activity, resulting in higher electron transport rates at high *p*CO_2_ and high irradiance, as well as higher assimilation rates. The gas exchange properties of AtpD-OE plants suggested that increased biomass and seed yield could be expected when plants are grown at high light and high *p*CO_2_. Further experiments including additional lines and targeted overexpression in leaves will clarify whether increasing the AtpD content presents a novel route for enhancing crop yield.

## Data Availability

Raw data and materials are available from the corresponding author upon request.

## Abbreviations

CEF: cyclic electron flow
Cyt*b*_6_*f*: cytochrome *b*_6_*f* complex
ECS: electrochromic shift
LEF: linear electron flow
NPQ: non-photochemical quenching
P700: reaction centre of PSI
*p*CO_2_: CO_2_ partial pressure
PGR5: PROTON GRADIENT REGULATION5
PhiNA: non-photochemical yield of PSI caused by acceptor side limitation
PhiND: non-photochemical yield of PSI caused by donor side limitation
PhiNO: yield of non-regulated non-photochemical reactions in PSII
PhiNPQ: yield of non-photochemical quenching
PhiPSI: effective quantum yield of PSI
PhiPSII: effective quantum yield of PSII
*pmf*: proton motive force
WT: wild type
